# Chronic Liver Disease and the Detection of Hepatocellular Carcinoma by [^18^F]fluorocholine PET/CT

**DOI:** 10.3390/diagnostics5020189

**Published:** 2015-05-19

**Authors:** Sandi A. Kwee, Linda L. Wong, Brenda Y. Hernandez, Owen T.M. Chan, Miles M. Sato, Naoky Tsai

**Affiliations:** 1 Hamamatsu/Queen’s PET Imaging Center, The Queen’s Medical Center, Honolulu, HI 96813, USA; E-Mail: kwee@hawaii.edu; 2 Cancer Center, The University of Hawaii, Honolulu, HI 96813, USA; E-Mails: hepatoma@aol.com (L.L.W.); brenda@cc.hawaii.edu (B.Y.H.); owenchan1@gmail.com (O.T.M.C.); 3 Affiliated Clinical Research Departments, The Queen’s Medical Center, Honolulu, HI 96813, USA; E-Mails: msato@queens.org (M.M.S.); naoky@hawaii.edu (N.T.)

**Keywords:** Positron Emission Tomography, hepatocellular carcinoma, fluorocholine

## Abstract

Positron emission tomography (PET) using the radiopharmaceutical tracer fluorine-18 fluorocholine (FCh) can elucidate tumors based on differences in choline phospholipid metabolism between tumor and surrounding tissue. The feasibility of detecting hepatocellular carcinoma (HCC) using FCh PET has been shown despite constitutively high parenchymal choline metabolism in the liver. Since HCC frequently develops in the setting of chronic liver disease, we comparatively evaluated FCh PET/CT between cirrhotic and non-cirrhotic patients with HCC to investigate the effects of hepatic dysfunction on tumor detection and the tumor-to-background ratio (TBR) of FCh uptake. FCh PET/CT was performed prospectively in 22 consecutive patients with HCC (7 newly diagnosed, 15 previously treated). Of these 22 patients, 14 were cirrhotic and 8 non-cirrhotic. Standardized uptake value (SUV) measurements were obtained by region of interest analysis of the PET images. Tumor FCh uptake and the TBR were compared between cirrhotic and non-cirrhotic patients. Liver lesions were confirmed to be HCC by biopsy in 10 patients and by Barcelona criteria in 4 patients. There was correspondingly increased liver tumor FCh uptake in 13/14 of those patients, and iso-intense tumor FCh uptake (TBR 0.94) in one non-cirrhotic patient with newly diagnosed HCC. FCh PET/CT also showed metastatic disease without local tumor recurrence in 2 previously treated patients, and was negative in 6 treated patients without tumor recurrence by radiographic and clinical follow-up. Tumor maximum SUV ranged from 6.4 to 15.3 (mean 12.1) and liver TBR ranged from 0.94 to 2.1 (mean 1.6), with no significant differences between cirrhotic and non-cirrhotic patients (SUVmax 11.9 *vs.* 12.2, *p* = 0.83; TBR 1.71 *vs.* 1.51, *p* = 0.29). Liver parenchyma mean SUV was significantly lower in cirrhotic patients (6.4 *vs.* 8.7, *p* < 0.05). This pilot study supports the general feasibility of HCC detection by FCh PET/CT. However, a broad range of tumor FCh uptake was observed, and lower liver parenchymal uptake of FCh was noted in cirrhotic patients as compared to non-cirrhotic patients. Incorporating tissue profiling into future liver imaging trials of FCh PET may help determine the molecular basis of the observed variations in tumor and hepatic FCh uptake.

## 1. Introduction

Hepatocellular carcinoma (HCC) is the third leading cause of cancer mortality worldwide [1]. It is also projected to become the third most lethal cancer in the United States, surpassing breast, prostate, and colorectal cancer by 2030 [2]. Since surgical resection is potentially curative but appropriate only for early-stage HCC [3], there is a need for better diagnostic tests to detect liver cancer before it metastasizes or becomes unresectable.

Conventional imaging diagnostics such as X-ray computed tomography (CT) and magnetic resonance imaging have made significant progress in detecting and characterizing liver tumors [4]. These tests rely primarily on structural-anatomic assessments as the basis of disease detection. With the emergence of precision medicine, it may also be useful to image liver tumors on the basis of molecular or metabolic traits, particularly if those traits can provide information on prognosis or treatment vulnerability [5,6,7].

Positron emission tomography (PET) takes advantage of molecular biologic changes to identify diseased tissue. Using the radiopharmaceutical tracer fluorine-18 fluorodeoxy-d-glucose (FDG), PET can detect a variety of cancers since malignancy often exhibits increased glycolysis even under aerobic conditions (*i.e.*, the Warburg effect) [1,6]. However, for reasons potentially related to balanced glucose utilization in liver tumors and liver tissue [8], FDG PET/CT has performed sub-optimally for detecting primary HCC, with diagnostic sensitivity estimated in the range of 50%–60% [9,10,11].

This pilot study evaluates fluorine-18 fluorocholine (FCh) as an alternative oncologic PET tracer for detecting HCC. Biochemically, FCh traces the first steps of choline phospholipid (*i.e.*, phosphatidylcholine) synthesis, allowing PET to visually and quantitatively discriminate tissues based on intracellular choline transport and phosphorylation [12]. Increased levels of choline-containing metabolites are present in many different types of cancer, which supports choline metabolism as a molecular imaging target for detecting tumors [13]. In clinical studies conducted at a single-institution, FCh has shown superiority over FDG for detecting primary HCC [11]. However, HCC often arises in the setting of chronic liver disease, raising the question of whether underlying liver function can influence primary tumor detection by FCh PET. To address this question, this pilot study evaluates HCC detection by FCh PET/CT in cirrhotic and non-cirrhotic patients, using the tumor-to-background ratio (TBR) of FCh uptake to base comparisons related to the severity of underlying liver disease.

## 2. Methods

### 2.1. Patients

Twenty-two sequential adult patients with a history of HCC diagnosis (7 newly diagnosed and 15 previously treated) were prospectively enrolled to this institutional review board approved U.S. single-institution study. Written informed consent was obtained from all patients prior to study participation. Clinical data collected by the study included patient demographics and information on HCC risk factors such as infection by hepatitis B (HBV) or hepatitis C (HCV), significant alcohol use (*i.e.*, at least 2 alcoholic beverages daily for 10 years), or other relevant underlying liver disorder. Data on tumor size, cancer stage, and prior treatment (partial hepatectomy, orthotopic liver transplant, and liver-directed therapy such as transarterial chemoembolization and radiofrequency ablation) were also collected.

### 2.2. Fluroine-18 Fluorocholine Synthesis and PET/CT Imaging

FCH synthesis was performed by fluorination of ditosylmethane with fluorine-18 followed by alkylation of the fluorotosylmethane intermediate with dimethylethanolamine using a chemical process control unit (CTI/Siemens CPCU, CTI/Siemens, Knoxville, TN, USA) [14]. All synthesis products passed standard assays for radiochemical purity, radionuclidic identity, chemical purity, and non-pyrogenicity prior to injection. Radiochemical purity was greater than 99%.

PET/CT imaging of the torso was performed using a Philips Gemini TF-64 PET/CT scanner (Philips Healthcare, Andover, MA, USA). A CT transmission scan was first performed in the supine position. The 64-channel helical CT scanning parameters were: 120 kV, 50 mA/slice, rotation time 0.75 s, slice thickness/interval 5.0 mm. No iodinated intravenous contrast was used for CT. At approximately 15 min following the intravenous injection of 2.2 to 3.0 MBq/kg of FCh, emission scans were acquired over multiple bed positions at 2-min per bed position. Image reconstruction employed a list-mode version of a maximum likelihood expectation maximization algorithm. The CT data was used for attenuation correction.

### 2.3. PET Image Analysis and Histologic Correlation

Potential liver lesions were identified and characterized on the basis of increased FCh uptake relative to adjacent normal tissue or organ background activity. The maximum standardized uptake value (SUV) of each lesion was recorded, with SUV defined as the maximum measured radioactivity from a region of interest (ROI) divided by the injected radioactivity normalized to body weight. A tumor to background ratio (TBR) was computed for all liver tumors by dividing the tumor maximum SUV by the average SUV corresponding to a 2 cm ROI placed in the liver parenchyma adjacent to the tumor ROI. While image interpretation for this pilot study was conducted without reader access to specific clinical information for each patient, the reader was aware that all patients either had newly diagnosed or previously treated HCC. In the event multiple liver tumors were detected, only the largest tumor was quantified to avoid bias towards patients with multiple tumors.

Histopathology was used as the primary standard of reference for confirming the diagnosis of HCC in this study. However, since it is not always clinically appropriate to perform liver biopsy for tumors meeting Barcelona Clinic Liver Cancer Group criteria for HCC, this criterion was also accepted for confirmation of HCC [15].

The diagnosis of liver cirrhosis was based on liver histology. For liver parenchymal SUV measurement, a standard 4 cm ROI was placed in the right hepatic lobe (or remnant lobe in cases of partial hepatectomy) distant from any tumor or previous treatment site. Measurements from this ROI were used to compare liver parenchymal uptake of FCh between cirrhotic and non-cirrhotic patients, since background liver tissue uptake adjacent to the primary tumor site could potentially be influenced by tumor mass effect or previous treatment.

Differences between groups were assessed by *t*-test. All tests were two-tailed with a *p* < 0.05 accepted as the threshold for statistical significance. Correlation was assessed by regression analysis. Statistical analysis was completed using SAS 9.2 (SAS Institute, Inc., Cary, NC, USA).

## 3. Results

The study included 13 male and 9 female patients with a mean age of 64 years (range 52 to 81 years). HCC risk factors included HBV infection in 7 patients, HCV infection in 8 patients, significant alcohol consumption in 1 patient, non-alcoholic steato-hepatitis in 2 patients, and porphyria cutanea tarda in 1 patient. In 3 patients, there were no identified HCC risk factors. Clinical demographics, cirrhosis status, Barcelona Clinic Liver Cancer (BCLC) staging classification, prior treatments, and PET/CT findings are summarized in Table 1.

Ten patients had liver tumors confirmed as HCC by biopsy and 4 patients by BCLC criteria. Among these 14 (7 newly diagnosed, 7 previously treated) patients with primary tumors confirmed at the time of PET imaging, 13 had tumors that demonstrated increased FCh uptake on PET imaging. Only one had a tumor that could not be distinguished visually, demonstrating iso-intense uptake with a SUV of 6.4 and a TBR of 0.94. This 5.7 cm tumor from a non-cirrhotic patient was well-differentiated (Edmondson-Steiner grade I) but exhibited a diffuse pattern of macrovesicular steatosis.

**Figure 1:**
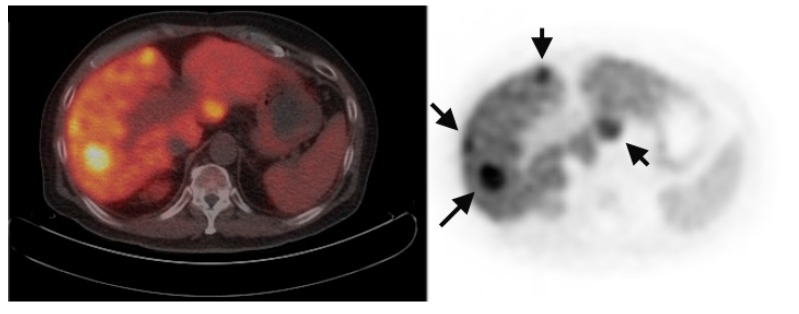
Multifocal recurrent hepatocellular carcinoma (HCC). Corresponding positron emission tomography (PET)/computed tomography (CT) (**left**) and PET (**right**) images demonstrate multiple foci of increased fluorocholine (FCh) uptake in the liver (*arrows*) ranging in diameter from 7 to 30 mm.

**Figure 2:**
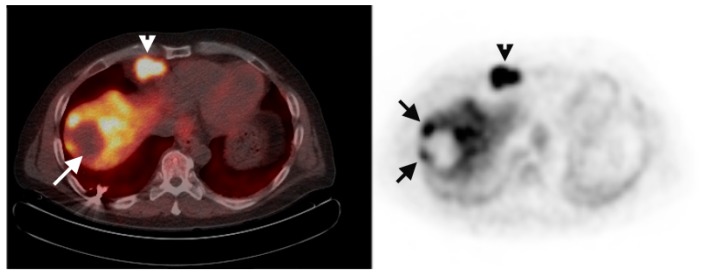
Recurrent HCC surrounding previous radiofrequency ablation site. PET/CT (**left**) shows absent FCh uptake (*white arrow*) in an area of the liver where HCC was previously treated by radiofrequency ablation. Corresponding PET (**right**) clearly shows multiple foci of increased FCh uptake surrounding the ablation site consistent with recurrent HCC (*arrows*). A pulmonary metastasis was also detected in this patient (*arrowheads*).

**Figure 3:**
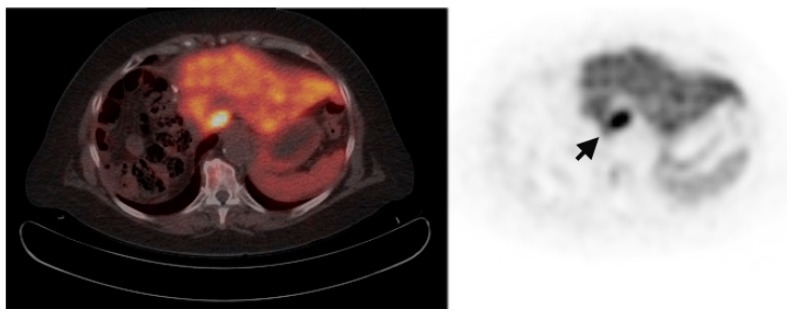
Tumor recurrence near a resection margin. Corresponding PET/CT (**left**) and PET (**right**) images of the remnant liver status post partial right hepatectomy shows focal increased FCh uptake (*arrow*) adjacent to the resection margin. Histology confirmed this lesion as recurrent HCC.

**Figure 4:**
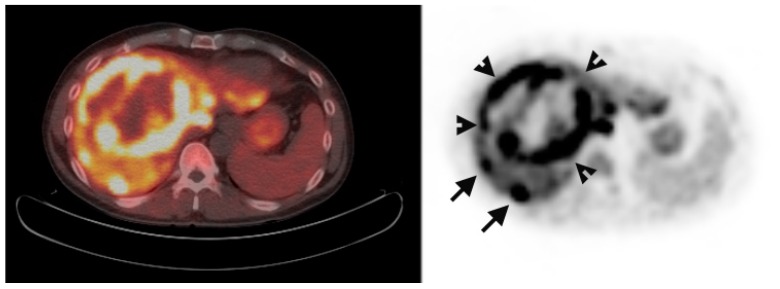
Metabolic heterogeneity in HCC. Corresponding PET/CT (**left**) and PET (**right**) shows a heterogeneous region of increased uptake within the liver in this patient with newly diagnosed liver mass. Two small satellite tumors (*arrows*) are adjacent to the dominant mass (*arrowheads surround*). Histologically, this was a poorly differentiated HCC tumor.

Multiple foci of increased FCh uptake in the liver consistent with multifocal or multinodular HCC (example, Figure 1) were noted in 8 of the 13 patients with increased primary tumor uptake. Of these, the areas of increased FCh uptake were adjacent to the treatment site (examples, Figure 2 and Figure 3) in 2 patients treated by local tumor ablation and 1 patient treated by liver resection. In one newly diagnosed case, PET demonstrated heterogeneous tumor FCh uptake with increased peripheral uptake and markedly diminished central uptake (Figure 4). Pathology in this case revealed a highly-necrotic tumor with Edmondson-Steiner grade 3 differentiation.

**Figure 5:**
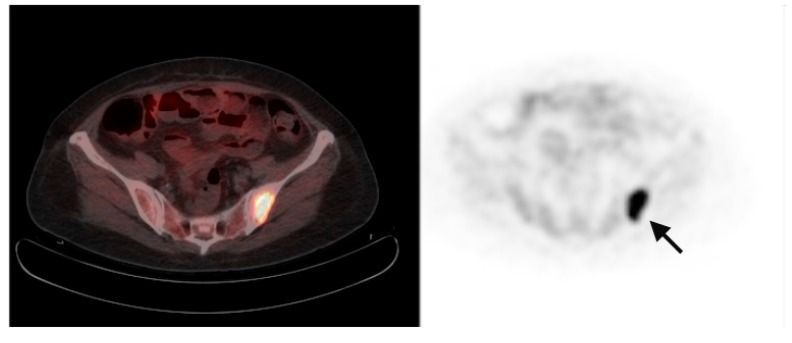
Metastatic HCC. Corresponding PET/CT (**left**) and PET (**right**) images show abnormal increased FCh uptake in the left posterior ilium (*arrow*) consistent with the diagnosis of skeletal metastasis.

The TBR corresponding to liver tumors ranged from 0.94 to 2.1 (mean 1.6). Corresponding tumor SUVmax ranged from 6.4 to 15.3 (mean 12.1). In 5 (1 newly diagnosed and 4 previously-treated) patients, PET demonstrated increased tissue FCh uptake outside of the liver. Corresponding clinical and radiographic findings in all these patients were consistent with metastatic disease. In 2 previously-treated patients, metastatic disease was seen on FCh PET without any evidence of primary liver tumor recurrence (example, Figure 5). In 6 patients without abnormal FCh uptake on PET, no evidence of recurrent or metastatic disease was noted after 6 months of clinical follow-up.

**Table 1:** Patient Characteristics and PET Findings.

ID	Age, Gender	Prior Tx (Initial BCLC Stage)	Cirrhosis	HCC Risk Factor	PET Liver Findings	PET Metastatic Findings	Max Liver Tumor Diameter in mm	Tumor SUVmax	TBR	Mean Liver SUV	Liver Tumor Confirmed by
1	59, M	OLT (A)	No	None	Multinodular Uptake	Abdominal nodes	30	14.3	1.65	7.7	Histology
2	65, M	RFA (A)	Yes	HCV	Multinodular Uptake	None	29	11.5	2.09	5.0	BCLC
3	55, F	PH (A)	No	HBV	Multinodular Uptake	None	23	15.3	1.10	12.4	BCLC
4	67, F	PH (A)	Yes	PCT	Uptake at resection margin	None	20	14.8	1.83	7.5	Histology
5	68, M	PH (A)	No	NASH	Multinodular Uptake	None	68	13.1	2.14	7	BCLC
6	60, M	RFA (A)	Yes	HCV	Uptake around treatment site	Lung and mediastinal nodes	25	11.1	1.49	6.6	BCLC
7	78, F	TACE (B)	Yes	HCV	Uptake around treatment site	None	70	11.5	1.58	6.0	Histology
8	64, F	OLT (A)	No	HBV	None	Lung and bone lesions	n/a	n/a	n/a	12.3	n/a
9	56, F	OLT (A)	No	HCV	None	Lung lesions	n/a	n/a	n/a	8.5	n/a
10	81, M	RFA (A)	Yes	HBV	None	None	n/a	n/a	n/a	7.7	n/a
11	80, M	RFA (A)	Yes	HCV	None	None	n/a	n/a	n/a	7.4	n/a
12	55, F	RFA (A)	Yes	HBV	None	None	n/a	n/a	n/a	6.5	n/a
13	61, F	RFA (A)	Yes	HCV	None	None	n/a	n/a	n/a	8.3	n/a
14	70, M	RFA (A)	Yes	Alcohol intake	None	None	n/a	n/a	n/a	4.7	n/a
15	52, M	RFA (A)	Yes	HCV	None	None	n/a	n/a	n/a	5.8	n/a
16	53, M	None (B)	No	HBV	Multinodular Uptake	None	125	11.2	1.56	6.7	Histology
17	65, F	None (A)	Yes	NASH	Solitary Uptake	None	49	13.9	1.49	7.2	Histology
18	79, M	None (B)	No	None	Solitary Uptake	None	152	13.0	1.68	8.8	Histology
19	60, M	None (B)	Yes	HBV	Multinodular Uptake	None	22	12.8	1.69	5.6	Histology
20	55, M	None (B)	Yes	HBV	Multinodular Uptake	None	24	10.9	1.55	7.2	Histology
21	78, F	None (B)	No	None	Iso-intense Uptake	None	57	6.4	0.94	6.1	Histology
22	54, M	None (C)	Yes	HCV	Multinodular Uptake	Mediastinal nodes	59	8.9	1.95	4.6	Histology

OLT = Orthotopic liver transplant; RFA = radiofrequency ablation; PH = partial hepatectomy; TACE = transarterial chemoembolization; NASH = Non-alcoholic steatohepatitis; BCLC = Barcelona Clinic Liver Cancer criteria; SUV = standardized uptake value; TBR = tumor-to-background ratio; PCT = porphyria cutanea tarda.

**Figure 6:**
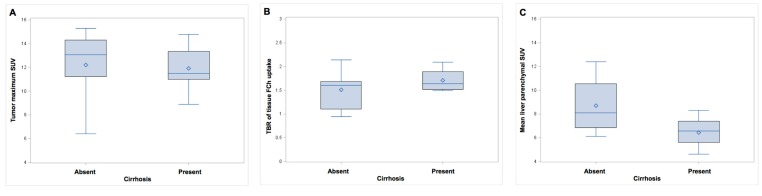
Box-plots of tumor maximum standardized uptake value (SUV) (panel **A**), tumor-to-background ratio (tumor-to-background ratio (TBR), panel **B**), and mean parenchymal liver SUV (panel **C**) in cirrhotic *vs.* non-cirrhotic HCC patients. Differences in tumor maximum SUV and TBR were not statistically significant. Mean SUV in the liver parenchyma of cirrhotic patients was significantly lower as compared to non-cirrhotic patients (6.4 *vs.* 8.7, *p* < 0.05). Upper and lower boundaries of boxes represent the 75th and 25th percentiles, respectively (Interquartile Ranges). Mean values are indicated by diamonds and median values are indicated by horizontal lines within the boxes.

Liver histology classified 14 patients as cirrhotic and 8 patients as non-cirrhotic. There was no statistically significant difference in mean TBR or tumor SUVmax between cirrhotic and non-cirrhotic patients (TBR 1.71 *vs.* 1.51, *p* = 0.29; SUVmax 11.9 *vs.* 12.2, *p* = 0.83). Cirrhotic patients demonstrated significantly lower liver parenchymal FCh uptake as compared to non-cirrhotic patients (mean parenchymal SUV 6.4 *vs.* 8.7, *p* < 0.05) (Figure 6). There was no statistically significant difference in parenchymal FCh uptake across HCC risk factors or between HBV and HCV infected patients. There was no significant correlation between greatest tumor cross-sectional diameter and tumor SUVmax.

## 4. Discussion

The results of this pilot study support the clinical feasibility of detecting primary HCC with FCh PET/CT in both cirrhotic and non-cirrhotic livers. The study results also support the potential for FCh PET/CT to detect metastatic disease as well as local tumor recurrence following treatment. Furthermore, the finding of a significant difference in liver FCh uptake between cirrhotic and non-cirrhotic patients raises the possibility of gauging liver dysfunction with FCh PET/CT. While studies in a broader spectrum of patients with chronic liver disease are needed, the possibility of using FCh PET to assess hepatocyte reserve may lead to expanded roles for imaging in risk stratification, pre-surgical planning, transplant candidate selection, and the monitoring of hepatotoxicity in systemically treated patients.

The first clinical study of FCh PET for detecting HCC was conducted by Talbot *et al.* [11]. Their initial study detected HCC in 12 of 12 patients who were imaged by FCh PET (8 newly diagnosed and 4 recurrent HCC) [11]. In 9 patients who also underwent conventional FDG PET imaging, only 5 of these patients had tumors detected on the basis of FDG uptake. A subsequent prospective study in 81 patients confirmed a superior rate of detection with FCh than with FDG, although data from this study also appears to suggest that FDG may have higher tumor specificity [16]. Like the current study, tumors smaller than 1 cm in diameter were detected with FCh PET/CT (Figure 1), which supports the potential for FCh PET to supplement existing radiographic criteria for making the non-histopathologic diagnosis of HCC.

The visual discrimination of tumors on PET relies on detecting differences in the intensity of uptake between malignant and benign tissue. Our finding of diminished parenchymal FCh uptake in cirrhosis raised the possibility that HCC may be more readily detectable in patients with chronic liver disease or cirrhosis. However, liver TBR was found to not differ significantly between cirrhotic and non-cirrhotic patients, suggesting that liver dysfunction may not necessarily increase liver tumor conspicuity. Furthermore, this study found no statistically significant difference in tumor maximum SUV between cirrhotic and non-cirrhotic patients. Overall, the results of this study are consistent with those from previous studies by Talbot *et al.* [11,16], indicating comparable detection of HCC across a range of underlying liver disease severity. Regardless, a larger multi-center clinical trial involving a broader clinical spectrum of patients will be needed to support clinical adoption of FCh PET/CT for liver tumor detection.

There are several limitations associated with this single-institution diagnostic study. Histopathologic data was not available for all tumor lesions given that a confirmatory biopsy was not clinically warranted in those patients with suspected tumor recurrence that met Barcelona criteria. There is also insufficient data from this preliminary study to determine diagnostic SUV thresholds with statistical confidence, or to estimate the overall diagnostic sensitivity and specificity of FCh PET/CT based on a small patient cohort. However, the data in Table 1 may be suitable for meta-analysis with other studies. Finally, this study includes both newly diagnosed and previously-treated patients with HCC. Since early diagnosis of HCC and evaluation of HCC recurrence are separate but equally important clinical problems, the current findings should be confirmed in larger studies evaluating FCh PET/CT in these specific contexts.

**Figure 7:**
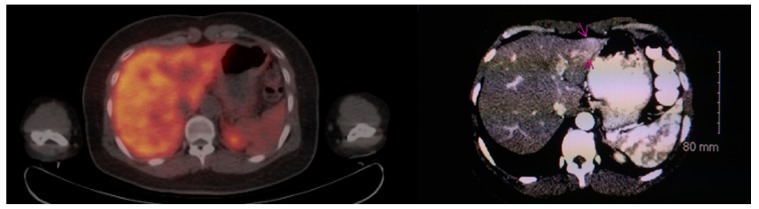
Primary HCC demonstrating iso-intense FCh uptake in non-cirrhotic patient. PET/CT image (**left**) shows no focal area of increased FCh uptake in the liver. However, a diagnostic CT scan with intravenous contrast (**right**) revealed a mass showing arterial enhancement and delayed washout in the left hepatic lobe lateral segment (*purple arrows*). Biopsy of this mass confirmed it to be a well-differentiated HCC.

This study shows that primary HCC can demonstrate a broad range of SUV values. This raises the possibility that FCh uptake is reflecting some phenotype of the tumor. Although a low TBR was encountered in a well-differentiated tumor (shown in Figure 7), other well-differentiated HCC tumors have been reported to show very high FCh uptake [16]. Clinical-translational studies involving tissue genomics or metabolomics could help to ascertain the molecular basis for these variations in tumoral and hepatic FCh uptake and potentially uncover novel biomarker applications in HCC.

## 5. Conclusion

Both HCC tumors and parenchymal liver tissue can demonstrate a broad range of FCh uptake, but the ratio of uptake between tumor and background does not appear to differ significantly between cirrhotic and non-cirrhotic patients. Differences in FCh uptake between cirrhotic and non-cirrhotic livers raises the possibility of applying FCh PET to gauge liver dysfunction. More studies on the underlying molecular biology of altered choline metabolism in HCC is needed to further clarify the potential clinical role of FCh PET/CT in this disease.
